# Refining and Assessing Gender-Tailored Group Therapy for Veteran Men With Military Sexual Trauma: Protocol for a Mixed Methods Study

**DOI:** 10.2196/105373

**Published:** 2026-09-01

**Authors:** Jonathan Yahalom, Erin P Finley, Sheila O'Brien, Brandon C Yarns, Lindsey L Monteith, Ariel J Lang, Alison B Hamilton

**Affiliations:** 1 Health Services Research, US Department of Veterans Affairs, 11301 Wilshire Boulevard, Los Angeles, CA, 90073, United States, 1 310 478 3711; 2 Department of Psychiatry and Biobehavioral Sciences, David Geffen School of Medicine, University of California, Los Angeles, CA, United States; 3 Central Texas Veterans Healthcare System, Temple, TX, United States; 4 VISN17 Center of Excellence for Research on Returning War Veterans, Waco, TX, United States; 5 VA Rocky Mountain Mental Illness Research, Education and Clinical Center for Suicide Prevention, Aurora, CO, United States; 6 Department of Psychiatry, University of Colorado Anschutz Medical Campus, Aurora, CO, United States; 7 Center of Excellence for Stress and Mental Health, US Department of Veterans Affairs, San Diego, CA, United States; 8 Department of Psychiatry, University of California, San Diego, CA, United States

**Keywords:** military sexual trauma, veteran men, group therapy, clinical trial design, treatment protocol

## Abstract

**Background:**

Military sexual trauma (MST) among veteran men is common, underreported, and associated with substantial psychological morbidity. Despite evidence that gender-related distress and shame pertaining to masculinity contribute to treatment avoidance and dropout, MST interventions within the US Department of Veterans Affairs have not been developed specifically for men.

**Objective:**

The objective of this study is to develop a gender-tailored group psychotherapy for veteran men with histories of MST. Guided by implementation science frameworks, the mixed methods study described in this paper proposes to iteratively refine the previously implemented Men’s MST Group (MMG), a 12-session, gender-tailored, trauma-focused group intervention that integrates dynamic group processes, mindfulness-informed distress tolerance, and psychoeducation.

**Methods:**

We draw on a synthesis of the intervention mapping framework and the access reconceptualization model to identify patient and system characteristics that contribute to treatment access and engagement. For aim 1, we will refine MMG through focus groups, qualitative interviews, and expert consultation. For aim 2, we will pilot-test MMG in a randomized controlled trial (n=32) comparing MMG to present-centered group therapy. For aim 3, we will evaluate implementation barriers and facilitators and iteratively refine the intervention through posttreatment qualitative interviews and expert panel review.

**Results:**

This study was funded with a start date of January 2025 and is anticipated to end in December 2029. As of July 2026, we have completed data collection for aim 1 and are currently analyzing data from 31 semistructured interviews. As of July 2026, we are also refining the MMG treatment protocol, which we plan to implement in a pilot randomized controlled trial in January 2027. Results of aim 1 are anticipated to be published in 2027. Participant recruitment for the aim 2 pilot trials has not begun.

**Conclusions:**

The goal of the current project is to refine and pilot-test a gender-tailored group psychotherapy for veteran men with histories of MST. We anticipate that the intervention will demonstrate feasibility, acceptability, and implementation potential because it was developed through an iterative process incorporating feedback from veterans, clinicians, and subject matter experts. Should study objectives be met, the next step will be to conduct a fully powered randomized controlled multisite trial. We additionally plan to disseminate findings regarding treatment needs, preferences, and barriers to care among men with histories of MST, which may inform future treatment development and implementation efforts beyond the current project.

## Introduction

### Background

Approximately 3.5% of veteran men and 44% of veteran women report a history of military sexual trauma (MST) [1]. Although women disproportionately experience MST, the absolute number of men who report MST (55,500 from 2004 to 2013) is relatively similar due to the larger proportion of men in the military [2,3]. Furthermore, MST likely remains vastly underreported among men, who experience multiple barriers to disclosure [4-7]. Men with histories of MST are also less likely to seek care compared to veteran women [2,6,8]. Thus, men with MST histories often wait decades to seek care for MST-related distress, contributing to pervasive life impairment [9-11]. While gender-based shame appears central to MST-related distress and treatment underuse, gender has often been overlooked in establishing evidence-based approaches, and to our knowledge, US Department of Veterans Affairs (VA) MST-related treatments have not been developed specifically for men [11].

To address this critical service gap, we drew on a time-limited, gender-tailored, and trauma-focused group therapy for men with histories of MST that was initially delivered as standard clinical care. The intervention (the Men’s MST Group; MMG) has been delivered for the past 7 years (2018-2025) and is designed to address the unique needs of this veteran population through promotion of agency, belongingness, and hope. Exploratory evaluation signaled strong retention as well as recovery and attitudinal shifts, including reductions in shame and posttraumatic stress disorder (PTSD) symptoms [12].

Rigorous research is needed to refine, test, and prepare for the implementation of this intervention. Moreover, given the fact that most men with histories of MST wait decades to seek treatment, research is needed to facilitate men’s use of MST-related services. In this research, guided by intervention mapping (IM) [13,14] and the access reconceptualization model (ARM) [15,16], we plan to iteratively refine the MMG intervention by interviewing veteran participants and mental health clinicians, conducting a pilot randomized controlled trial (RCT), and evaluating feasibility within the VA, with the long-term goal of national implementation. The goal of this paper is to describe the research protocol for this study.

### Men With Histories of MST: The Importance of Identity and Shame

MST is associated with transdiagnostic complexity and increased prevalence of PTSD and depression [17,18], substance use disorders [19], suicide [20,21], difficulty maintaining relationships and employment [2,3,22], and homelessness [23]. Men with histories of MST merit particular attention because core components of their distress may be underaddressed and undertreated. Research shows that men can often experience MST as an assault to masculine identity—holding erroneous beliefs that “if I had been more of a man I could have prevented the assault”—which can lead to profound ruptures in selfhood and avoidance [4,10]. Moreover, MST also has the potential to intersect with other aspects of one’s identity, including race, culture, and personal background—with perceptions that “they did this because of who I am”—thereby increasing one’s sense of shame [10,11].

In general, norms that define masculinity—stoicism, self-reliance, and suppression—are known to contribute to barriers to mental health treatment and recovery [24]. In the case of men’s MST, despite heightened distress, this population shows more pronounced patterns of treatment avoidance, underuse, and dropout. Male veterans with MST histories are no more likely to engage in mental health treatment than those without [8]. Moreover, male veterans use less MST-specific care than female veterans and are more likely to seek help for medical rather than psychiatric concerns [7]. Finally, while dropout rates from evidence-based practices, specifically prolonged exposure therapy (PE) and cognitive processing therapy (CPT) in VA settings, approach 40% for both men and women [25], individuals with MST as their focal trauma show difficulty participating in treatment [26].

**Figure 1.**
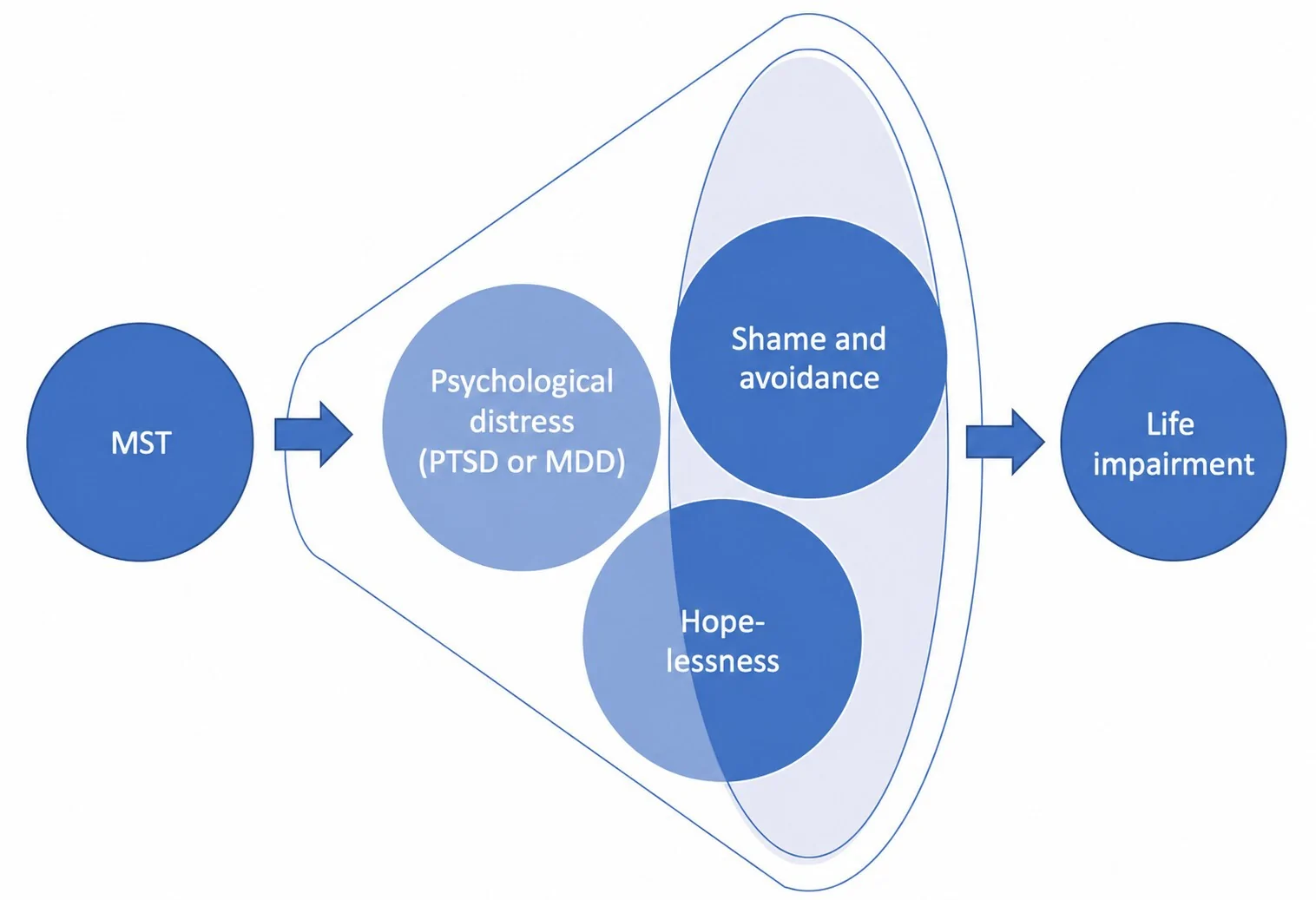
A model of untreated men’s military sexual trauma (MST). MDD: major depressive disorder; PTSD: posttraumatic stress disorder.

Shame and treatment underuse mean that male survivors of MST often wait decades to seek care, potentially contributing to chronic and pervasive life impairment [7]. Persistent psychological distress and avoidance (which can be due to shame) also contribute to loss of agency and hopelessness [27-31]. As shown in Figure 1, MST can simultaneously lead to psychological distress, shame and avoidance, and hopelessness, all of which can cause life impairment. Thus, more research is needed to understand how identity-related distress and shame contribute to treatment avoidance among men with MST and develop and evaluate strategies that improve treatment engagement, retention, and recovery outcomes.

### Current Treatment Standards

Current treatment standards for addressing distress pertaining to men’s MST are based on limited research and adapted from trauma-focused studies and, thus, may not sufficiently address concerns about identity and shame.

CPT and PE are two first-line therapies for PTSD endorsed by the VA for addressing MST-related distress [32]. While there is support for the use of both CPT [33-35] and PE for men with MST histories [36,37], additional options for treatment may be necessary for a number of reasons. First, PTSD symptoms among veterans completing these treatments often remain above clinical cutoffs, and many patients recover to a degree that they no longer meet PTSD diagnostic criteria [38-40]. While this finding is not unique to CPT and PE, this suggests a need for treatment refinement and augmentation of existing trauma-focused treatments, including development of novel treatment approaches [37]. Second, CPT and PE also have high dropout rates. Approximately 38% to 40% of PE and CPT participants at the VA terminate treatment prior to completion, reflecting the difficulty of overcoming issues of engagement with treatment [25,26]. Thus, while dropout may be due to the nature of avoidance associated with both PTSD and trauma exposure, trauma-focused treatments may be particularly challenging for survivors of MST [6,8]. Third, these interventions are gender inclusive (not gender tailored) despite evidence that gender tailoring psychological interventions may be a useful strategy to enhance treatment engagement and outcomes [41]. Moreover, CPT and PE are trauma specific and not transdiagnostic; they assume a model where fear and anxiety are the core emotional components of MST-related distress, which can lead to underaddressing gender-based identity [42].

In addition to CPT and PE, two specialized group protocols have been promoted to address MST: Warrior Renew [43-45] and Courage Group [46]. Both are gender inclusive, which means that they are designed to offer relevant treatment to both men and women, can be delivered with mixed-gender cohorts, and promote group discussion that is likely significant to overcoming isolation and shame among male survivors of MST. However, because neither protocol is gender tailored specifically for men, more research is needed both for group MST-focused approaches and men’s MST treatment more generally.

### Gender-Tailored Group Psychotherapy: The MMG

Drawing on these observations, we propose to study an alternative treatment approach that is gender tailored, trauma informed, and transdiagnostic.

Group therapy is promising for several reasons. First, group therapy may be relevant for individuals with heightened shame and isolation who, in a group setting, may experience relief and belonging by being with peers with shared experiences [47-49]. Second, group therapy can rebuild masculine belongingness through mutual recognition and development of social support [50]. Third, group participation and orientation to helping fellow veterans has potential to leverage a military ethos of brotherhood and service [51].

The MMG is a trauma-focused and discussion-based group tailored to men with MST [9,12]. The group is informed by an acceptance-based and psychodynamic treatment framework that emphasizes mindful awareness of internal experience, affective tolerance, and the exploration of relational and intrapsychic meanings of trauma within a supportive group context. The group runs for 90 minutes and, with the exception of the first session, which serves to orient new cohorts, is structured with the following sequencing: (1) mindfulness practice (5-15 minutes); (2) open group discussion of a theme pertaining to MST recovery (30-45 minutes); (3) invitation for a group member to discuss an MST event, with encouragement to disclose emotionally laden memories of the trauma, followed by a dynamically oriented group discussion (30 minutes); and (4) coping skill practice (3 minutes).

A recent study of the MMG found initial evidence on its feasibility, acceptability, and clinical benefit [12]. Feasibility was reflected in high levels of engagement across the treatment pathway as over 80% of referred veterans attended at least one session and over 80% of those who began treatment remained until completion. Acceptability was reflected through qualitative data that showed that most patients described positive experiences of the group, including social connectedness, diminished shame, enhanced emotion regulation, greater engagement in treatment, and a renewed sense of hope. Clinical benefit was indicated through a medium–effect size change from before to after treatment in PTSD symptoms (Cohen *d*=0.666) and improved recovery (Cohen *d*=0.607) and a small–effect size change in shame and belongingness.

Given these initial findings, we propose to iteratively refine the MMG by interviewing veteran participants and mental health clinicians, conducting a pilot RCT, and evaluating feasibility within the VA.

## Methods

### Research Design

**Figure 2.**
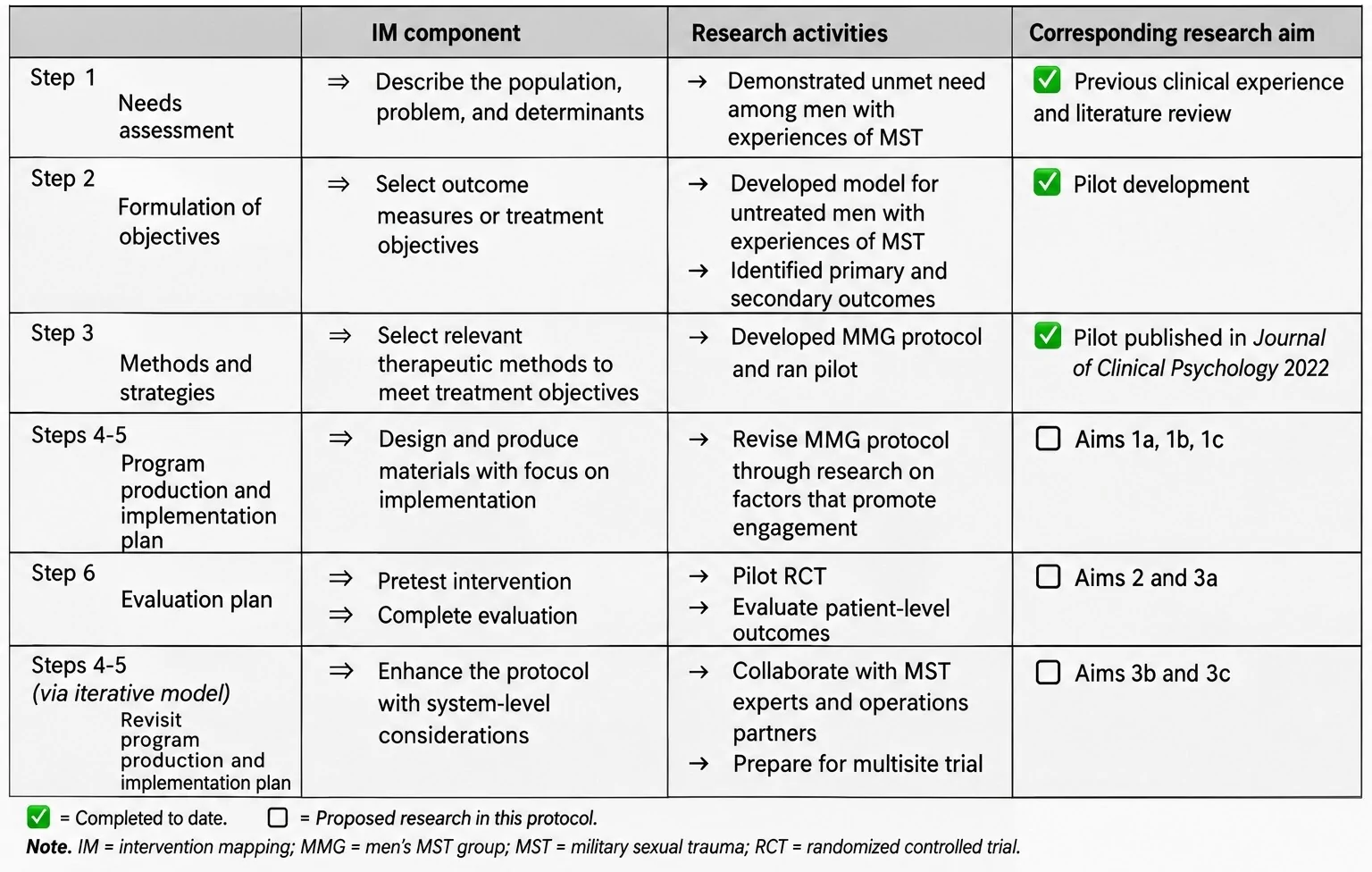
An intervention mapping (IM) approach to the Men’s Military Sexual Trauma (MST) Group (MMG). RCT: randomized controlled trial.

This protocol was developed and reported in accordance with the SPIRIT (Standard Protocol Items: Recommendations for Interventional Trials) 2025 guidelines [52]. The research design and methods used in this study are informed by a synthesis of the IM framework and the ARM to identify patient and system characteristics that contribute to treatment access and engagement. IM is a sequential, iterative framework for planning, developing, and implementing evidence-based interventions, where the completion of one IM step results in a product that is the guide for the subsequent step [53]. In Figure 2, research activities are guided by relevant IM steps 4 to 6 (with steps 1-3 already completed).

### Setting

The proposed study will be conducted at the mental health clinic of the VA Greater Los Angeles (VAGLA), where over 4000 unique veterans receive care annually. All interviews will be conducted via Microsoft Teams or in person in a private clinical office. Treatment will be conducted in person in a private VA clinical setting.

### Ethical Considerations

This protocol has been approved by the VAGLA Institutional Review Board (IRB). The trial is registered at ClinicalTrials.gov (NCT06741202). All study procedures will be conducted in accordance with the ethical standards of the IRB and applicable regulations.

### Aim 1: Treatment Refinement

#### Overview

To revise the MMG treatment protocol with attention to factors that support men’s access to and engagement and retention in mental health interventions (IM steps 4-5), we will pursue 3 subaims. For aim 1a, we will investigate factors related to men’s use of MST-related services by conducting semistructured qualitative interviews (n=24) with VA mental health clinicians, Vet Center clinicians, MST coordinators, and MST experts. Results from aim 1a will inform revision of the protocol. For aim 1b, we will review the revised protocol by convening 2 focus groups with the 2 most recent MMG cohorts (n=8). For aim 1c, we will convene a subset of aim 1a participants for an expert panel to finalize the MMG protocol.

#### Data Collection

For aim 1a, we will conduct individual 30- to 45-minute semistructured interviews virtually with clinicians and other experts. Participants will be asked about factors related to men’s mental health treatment engagement, including (1) strategies for promoting access and engaging and retaining male survivors of MST in VA-based treatment, (2) strategies for engaging male survivors of MST in a gender-tailored group therapy, (3) suggestions for improving MMG treatment components and group discussion topics, and (4) strategies for addressing gender and intersectional factors in psychotherapy for veteran men with MST histories. For aim 1b, we will conduct 2 virtual focus groups lasting approximately 60 minutes each and focus on MMG revisions. For aim 1c, we will conduct an MST expert panel comprising individuals (approximately 15) drawn from the 24 individuals who participated in aim 1a interviews and who expressed willingness to be on the expert panel. Operations partners will also be invited. Participants will be asked to advise on engagement strategies, MMG core components, and outcome measures. Subject matter expert diversity will be achieved through invitations to participate based on representation of clinician types [54].

#### Analysis

Data will be analyzed using rapid qualitative analysis (RQA), an established, pragmatic, team-based approach that quickly generates preliminary findings to inform real-time modifications and processes [55]. RQA involves (1) creating at least one neutral domain name (ie, topic) for each interview question; (2) creating a summary template with the domains to facilitate data condensation, in which the key data points are summarized; (3) testing the template for its utility and for establishing group process; (4) after verifying template utility and group process, completing a templated summary of each interview; (5) transferring summary points to a data matrix; and (6) conducting matrix analyses to assess the content of interview domains and any gaps in information and developing summaries of themes. Matrices will be developed for data from aims 1a and 1b and will be triangulated with regard to recommended revisions to the MMG protocol. Learnings from preresearch focus groups and the MST expert panel will be incorporated into intervention revisions and used to develop a comprehensive intervention description informed by the Template for Intervention Description and Replication (TIDieR) checklist [56].

### Aim 2: Pilot Trial

#### Overview

Using the revised protocol, we will conduct a pilot RCT of the MMG vs present-centered group therapy (PCGT; control condition) at an outpatient VAGLA mental health clinic (IM step 6). Over 24 months, male survivors of MST (n=32) will be randomized to the MMG or PCGT group (4 cohorts: 2 experimental and 2 control). We will investigate engagement (session attendance) as well as recovery-oriented outcomes (agency, hope, belongingness, and shame reduction) and psychological symptoms (PTSD, major depressive disorder, and suicidality) assessed before, immediately after, and 6 months after the intervention.

#### Participant Recruitment and Randomization

We will begin recruiting male survivors of MST through collaboration with VAGLA outpatient mental health clinics (ie, primary care, mental health clinics, trauma recovery services, substance use disorder clinics, and domiciliary services). During standard clinical care, health care professionals will inform male survivors of MST about the opportunity to participate in a study on men’s MST. These veterans will receive an informational study handout, which will also be publicly available. This handout will state that veterans will potentially receive financial compensation for baseline data collection. Interested veterans will be screened for eligibility and contact the study coordinator to provide written consent and arrange the collection of pretreatment data.

Individuals who do not meet the exclusion criteria (see below) will be randomly assigned to 1 of the 2 study groups: the MMG (n=8) or PCGT control (n=8). The process will be repeated for the second cohort. Participant randomization will be performed using a computer-generated randomization sequence prepared by an independent statistician, with allocation concealment maintained until assignment. Due to the nature of the intervention, participants cannot be blinded following treatment assignment. Because primary outcomes are patient reported, blinded outcome assessment is not feasible.

#### Data Management

All qualitative and quantitative data will be linked to a unique identification number assigned to each participant and entered into computerized files. Only the identification number will be linked to participant data in databases and paper documents. We will secure all paper records and electronic data files in compliance with current VA IRB policies. All data will be coded by the study coordinator. Datasets will be maintained for screening for out-of-range values and comparing a random sample of 10% of entered data to the original data collection forms. Problems or perceived errors with data entry will be addressed iteratively. Double entry procedures will be used on a select (10%) sample to assess for errors.

#### Eligibility Criteria

Eligible participants include any individual who identifies as a veteran cisgender man with a history of MST, speaks English, is aged 18 years or older, and is enrolled in VAGLA. Participants must (1) comprehend and sign the informed consent form, (2) report a history of MST, and (3) agree to complete the research instruments. Because this pilot trial is primarily designed to assess for feasibility, acceptability, and clinical signal, concomitant care (eg, psychiatric or individual and group therapy) will be permitted.

#### Exclusion Criteria

Veterans will be excluded if they demonstrate (1) severe suicidal ideation defined using the Columbia-Suicide Severity Rating Scale, chart-documented homicidal ideation, and behavioral flags per chart review; (2) severe and uncontrolled substance abuse (identified through chart review and discussion with health care professionals); (3) illness that inhibits engagement in study procedures (eg, inability to attend in-person visits); (4) inability to self-consent to participate; (5) completion of the MMG 5 or fewer years before; and (6) concurrent enrollment in a trauma-focused intervention. Through chart review, veterans who drop out of MMG or trauma-focused interventions after 3 sessions or less will still be eligible as this is considered early dropout. If immediate clinical attention is warranted, veterans will receive care via procedures outlined in the human participant protection plan.

#### Design

##### Overview

This pilot RCT will involve 12 weekly in-person sessions with an interventionist at the West Los Angeles Mental Health Clinic. The principal investigator (PI) will serve as the primary interventionist for the experimental condition during the first cycle, and a different staff psychologist will serve as the interventionist for the experimental condition during the second cycle. To distribute and minimize potential health care professional burden, 2 additional staff psychologists from Trauma Recovery Services Clinic (1 per cycle) will serve as interventionists for the control condition. All group sessions will be audio recorded for fidelity. Participant retention will be enhanced through financial compensation for trial participation. Participants will receive US $100 for completion of a pretreatment survey, US $50 for completion of the posttreatment survey, and US $50 for completion of the 6-month survey and interview.

While having the PI conduct the first experimental condition risks bias, this was strategically designed for three key reasons: (1) the PI developed the MMG treatment protocol and, thus, is most familiar with the approach; (2) it will allow for any needed protocol adjustments in preparation for training of another clinician for the second cohort; and (3) this will be a pilot RCT designed to assess appropriateness and feasibility for a future trial.

##### MMG (Experimental Condition)

This is a 12-week group that meets for 90 minutes weekly. This group will draw on interventions developed in our pilot study (described above). The protocol will be further revised based on IM steps followed for aims 1 and 2.

##### PCGT (Control Condition)

This is a time-limited treatment for PTSD that focuses on increasing adaptive responses to current life stressors and difficulties that are directly or indirectly related to trauma or PTSD symptoms. PCGT was initially developed as a nonspecific comparison condition to test the effectiveness of trauma-focused cognitive behavioral therapy [57]. To justly compare it to MMG, PCGT will be delivered for a 12-week period. The basis of PCGT is to provide “common factors” of psychotherapy and help articulate whether the effect of MMG is due to bringing men together in a group setting or delivering treatment that is specifically oriented to men (such as MMG).

### Data and Outcome Measures

The following primary and secondary outcomes will be measured at baseline, treatment completion, and 6-month follow-up.

#### Primary Exploratory Outcomes

The Recovery Assessment Scale (RAS) is a 41-question forced-choice 5-level Likert rating scale designed to assess recovery, with emphasis on agency, hope, belongingness, and shame reduction—key constructs postulated to impact and be expressive of psychological distress and intra- and interpersonal avoidance [58]. The External and Internal Shame Scale (EISS) assesses global shame experiences, with attention to its external and internal dimensions relevant to men’s MST (Figure 1) [59]. The scale consists of 8 items and measures 4 central shame domains: inferiority and inadequacy, sense of exclusion, uselessness and emptiness, and criticism and judgment. The General Belongingness Scale (GBS), a 12-item measure rated on a 7-point Likert scale, assesses a general sense of belonging, another indication of interpersonal distress pertaining to MST [60].

#### Secondary Exploratory Outcomes

The PTSD Checklist for DSM-5 (PCL-5) is a 20-item non–MST-specific self-report measure that assesses the *Diagnostic and Statistical Manual of Mental Disorders*, 5th edition, symptoms of PTSD, a common indicator of MST-related distress [61]. Each symptom is rated on a 5-point Likert scale assessing distress. The Patient Health Questionnaire–9 (PHQ-9) is a widely used brief, 9-item instrument for screening, monitoring, and measuring the severity of depression over the course of 2 weeks. The PHQ-9 is a reliable and valid measure of depression severity that assesses symptoms, including suicidality, that are consistent with the *Diagnostic and Statistical Manual of Mental Disorders*, 5th edition, criteria for depression [62].

### Analysis

Following the CONSORT (Consolidated Standards of Reporting Trials) extension for randomized pilot and feasibility trials [63], the limited dataset will be underpowered to detect differences between conditions, and we did not design this pilot RCT to have power to examine or establish efficacy; rather, we are seeking to pilot the refined intervention to assess engagement outcomes, change in theorized targets, and initial clinical signals. Any missing data will be identified during analysis and noted in write-ups. We will use a linear mixed model for longitudinal data with a 2-level factor for group and a 3-level factor for time. The experimental and control groups will form the 2-level between-group factor, and the 3 time points (baseline, after treatment, and 6-month follow-up) will form the within-group factor.

To pretest the intervention, we will test the experimental vs control groups on exploratory data on clinically meaningful outcomes from baseline to after treatment. We hypothesize that participants assigned to the MMG will report improved engagement (session attendance) and comparable signs of recovery (agency, hope, belongingness, and shame reduction) and PTSD, depression, and suicidality measurements. This hypothesis will compare the impact of the MMG to that of PCGT at the posttreatment time point and 6-month follow-up (both as compared to baseline). Both comparisons will include psychometrically validated measures (RAS, EISS, GBS, PCL-5, and PHQ-9) and attendance.

### Aim 3: Evaluation

#### Overview

To evaluate the design and complete an evaluation plan (IM step 6), we will assess pilot RCT findings and plan for efficacy and mechanistic trials. To do so, we will pursue 3 subaims. For aim 3a, we will conduct semistructured interviews with RCT participants (including completers, dropouts, and individuals who declined participation) and interventionists to understand treatment engagement factors, as well as perceptions of feasibility, acceptability, appropriateness, and effectiveness. For aim 3b, we will reconvene the aim 1c expert panel to discuss RCT results and potential facilitators, barriers, and strategies for implementation of the MMG in usual VA mental health care. For aim 3c, we will design the intervention package and enhance the protocol with system-level considerations (following a synthesis of the iterative IM and ARM frameworks by returning to IM steps 4-5). Insights gathered from aims 3a, 3b, and 3c will contribute to final enhancements to the MMG protocol.

#### Data Collection

Veterans who are screened as eligible but decline to enroll (n≈7) will be invited to participate in a brief (5-20 minute) interview to understand their reasons for declining and their history (if any) of use of and experiences with MST-related services and mental health services more broadly to align with the ARM. In addition, those who enroll but drop out before treatment completion (n≈7) and all completers (n≈16) will be invited for 20- to 45-minute interviews. All interviews will be arranged within a reasonable period. We estimate interviewing a total of 30 veterans, with the assumption that approximately half of RCT completers will not participate in interviews due to attrition, lack of interest, and other unforeseen barriers. We will intentionally recruit a diverse sample of participants in terms of racial and ethnic identities, ages, and sexual orientations to explore intersectionality. All veterans who participate in interviews will receive financial compensation.

Semistructured interviews with interventionists will solicit information regarding (1) clinician perceptions of men’s engagement; (2) quality and effectiveness of the intervention; and (3) quantitative data on acceptability, appropriateness, and feasibility of the interventions based on brief 4-item measures (Acceptability of Intervention Measure, Intervention Appropriateness Measure, and Feasibility of Intervention Measure) [64].

#### Analysis

Analysis of aim 3a interview data will use both RQA (for process outcomes) and inductive thematic analysis (for person-centered components). Thematic analysis emphasizes identifying, analyzing, and interpreting patterns of meaning within qualitative data [65]. Using ATLAS.ti (ATLAS.ti Scientific Software Development GmbH), transcripts will be reviewed, and segments of narratives will be tagged (ie, coded) by semantic clusters. We will perform the analysis to categorize semantic meaning, and segments will be assigned more than one code if they convey multiple meanings. Codes will be grouped to generate emergent themes, defined as coalescing patterns that signify broader semantic meaning. We will also attend to themes relevant to the interview topics and to divergent narratives to further saturate understanding.

Aim 3a qualitative data will be triangulated with quantitative data from aims 2 and 3 and chart review data. This mixed methods analytic plan will be used to generate further evaluation of the intervention (IM step 6). Quantitative data collected from the RCT are meant to assess specific facets of treatment engagement, psychological distress, impairment in recovery, and shame and belonging, which will be used to provide complementary and contrasting perspectives on the qualitative data gathered for aim 3a. To evaluate the intervention using integrated mixed methods, treatment targets and outcome measures will be mapped to specific modes of treatment used in the MMG (eg, mindfulness, discussion topics, and coping skill education). These results will be presented to the expert panel for aim 3b.

#### Expert Panel and Protocol Enhancement

We will reconvene the aim 1c expert panel to discuss RCT results and potential facilitators of and barriers to implementation of the MMG in general and in different organizational contexts (eg, mental health services in large vs small VA medical centers vs community-based outpatient clinics). We will specifically engage MST experts to understand pilot RCT results and gain contextualization of these results to engage, to respond to, and to fill gaps in MST-related mental health services. We will also seek to leverage data from this project to develop recommendations to improve strategies to engage men in pursuing MST-related care earlier in their lifetimes.

During meetings with operations partners, we will design the intervention package and enhance the MMG protocol through discussing results from aims 3a and 3b with a focus on identifying system-level considerations and assessing the fit of the MMG in usual mental health care, as well as potential strategies that would support implementation. This will facilitate preparation for efficacy and mechanistic trials to detect main treatment effects and mixed methods evaluations to explore preliminary implementation outcomes such as adoption, acceptability, feasibility, and sustainability to prepare for subsequent implementation trials.

## Results

This study was funded with a start date of January 2025 and is anticipated to end in December 2029. As of July 2026, we have completed data collection for aim 1 and are currently analyzing data from 31 semistructured interviews. As of July 2026, we are also refining the MMG treatment protocol, which we plan to implement in the pilot RCT in January 2027. Results of aim 1 are anticipated to be published in 2027.

## Discussion

Men with histories of MST are an important yet underserved veteran population. Shame and disruptions related to masculine identity may contribute to substantial distress and treatment underuse, highlighting the need for research that addresses the specific clinical needs of this population. Existing treatments are valuable but were not developed specifically to address men’s MST-related identity concerns. This study addresses that gap by refining and piloting the MMG, a group psychotherapy intervention designed specifically for men with histories of MST and that has demonstrated previous preliminary clinical benefit. We anticipate that this pilot will provide preliminary evidence regarding the feasibility, acceptability, and potential clinical utility of this gender-tailored group intervention.

The protocol has several methodological strengths. First, the iterative intervention refinement approach guided by the IM and ARM frameworks incorporates perspectives from veterans, clinicians, and an MST expert panel. Second, this protocol is designed to examine key implementation factors and for future implementation considerations by exploring engagement, dropout, barriers, and facilitators. Third, the mixed methods integration provides a richer understanding of feasibility and preliminary clinical effects, where quantitative outcomes can characterize clinical benefit and qualitative data can explore context and acceptability, which will be useful for future research on men with histories of MST.

Several limitations should be considered when interpreting findings from this pilot study. First, the pilot sample size, designed for feasibility and clinical signal to inform future research, is underpowered for efficacy testing. Second, this protocol will be conducted at a single site, and implementation may vary across VA facilities. Third, there is potential for selection bias given that men willing to participate in a group therapy trial for MST may differ from a more representative sample of men with MST, who may be less likely to seek MST-focused care and participate in group interventions. Fourth, therapist effects may influence outcomes, particularly given that the intervention was initially delivered and will be tested by the PI; fidelity monitoring and standard intervention procedures will help mitigate this concern.

Findings of this study will be disseminated to describe treatment needs, preferences, and barriers to care for men with histories of MST, which may inform future treatment development and implementation efforts beyond the current project. If the pilot demonstrates feasibility and identifies a viable implementation pathway, the next step will be to conduct a fully powered multisite trial to evaluate intervention effectiveness while exploring implementation outcomes.

## Supplementary material

10.2196/105373 Peer Review Report 1 Peer review report by the VA Office of Research and Development (ORD).

## Abbreviations

ARM: access reconceptualization model
CONSORT: Consolidated Standards of Reporting Trials
CPT: cognitive processing therapy
EISS: External and Internal Shame Scale
GBS: General Belongingness Scale
IM: intervention mapping
IRB: Institutional Review Board
MMG: Men's Military Sexual Trauma Group
MST: military sexual trauma
PCGT: present-centered group therapy
PCL-5: PTSD Checklist for DSM-5
PE: prolonged exposure therapy
PHQ-9: Patient Health Questionnaire–9
PI: principal investigator
PTSD: posttraumatic stress disorder
RAS: Recovery Assessment Scale
RCT: randomized controlled trial
RQA: rapid qualitative analysis
SPIRIT: Standard Protocol Items: Recommendations for Interventional Trials
TIDieR: Template for Intervention Description and Replication
VA: United States Department of Veterans Affairs
VAGLA: United States Department of Veterans Affairs Greater Los Angeles
