# Supplementary material for: Refining and Assessing Gender-Tailored Group Therapy for Veteran Men With Military Sexual Trauma: Protocol for a Mixed Methods Study
Source: JMIR Res Protoc. 2026 Sep 1;15:e105373. doi: 10.2196/105373 (PMC13533415; doi:10.2196/105373)
Supplement: Supplementary file 1 [file resprot-v15-e105373-s001.pdf]

SUMMARY STATEMENT
(Privileged Communication)

PROGRAM CONTACT:
Robert Small
(202) 443-5743
robert.small@va.gov

Release Date: **09/27/2023**
Revised Date:

Application Number: **1IK2HX003783-01A1**
Formerly: **1IK2HX003783-01**

Principal Investigator

YAHALOM, JONATHAN

Applicant Organization: VA GREATER LOS ANGELES HEALTHCARE SYSTEM

Review Group: **MRA0**
Mentored Research

Meeting Date: **08/22/2023**
Council: **OCT 2023**
Requested Start: **01/01/2024**

Opportunity Number: **HX-23-009**
PCC: **HXSMALL**

Project Title: **Recovery-oriented group therapy for Veteran men with military sexual trauma**

SRG Action: **Impact Score:183**

Human Subjects: 30-Human subjects involved - Certified, no SRG concerns
Animal Subjects: 10-No live vertebrate animals involved for competing appl.
Gender: 1A-Both genders, scientifically acceptable
Minority: 1A-Minorities and non-minorities, scientifically acceptable
Age: 1A-Children, Adults, Older Adults, scientifically acceptable
Clinical Research - not NIH-defined Phase III Trial

Project Year	Direct Costs Requested
1	193,228
2	195,910
3	199,695
4	163,079
5	166,340
TOTAL	918,252

ADMINISTRATIVE BUDGET NOTE: The budget shown is the requested budget and has not been adjusted to reflect any recommendations made by reviewers. If an award is planned, the costs will be calculated by VA Office of Research and Development (ORD) staff based on the recommendations outlined in the BUDGET COMMENT section and any relevant ORD service-specific limitations.

KEY SUMMARY POINTS:

1. Outstanding candidate who leverages his clinical background in the service of a proposed health services research career. Well-aligned mentoring team and local research and operations support.

2. Very comprehensive response to prior critiques.

3. The conceptual framework does not appear to adequately encompass several key areas of relevance to the proposal, especially patient and system characteristics that contribute to access and engagement in treatment for MST by men.

4. The feasibility of recruiting patient participants for Aim 1 and collecting the focus group data as proposed is not convincing.

5. Movement to a Hybrid Type 1 effectiveness study in Year 5 seems premature given the inability to test efficacy in *any* pilot, and the short timeline between the Year 3 and year 5 Merits is a concern.

DESCRIPTION (provided by applicant):
Background: Military sexual trauma (MST) is associated with significant morbidity – including PTSD and depression, substance use, suicide, and medical comorbidities – yet men who have experienced MST are known to underutilize treatment. Gender-based stigma and shame, distrust of the VA, and the debilitating impact of trauma likely all contribute to treatment underutilization among men MST survivors. Gender-tailored care for this population holds the potential for earlier and more sustained engagement in care. The Men's MST Group (MMG) was developed and delivered by the applicant with this prospect in mind. In this study, Intervention Mapping will guide the process of refining, testing, and evaluating the MMG, which has shown promise in engaging Veterans, promoting recovery, and reducing trauma symptoms. Significance/Impact: Approximately 3.5% of Veteran men (and 44% of Veteran women) report a history of MST, yet the absolute number of men who report MST (55,500 from 2004-2013) is relatively similar due to the larger proportion of men in the military. Due to its association with numerous deleterious health impacts, MST remains one of the VA's highest commitments, yet health services research on men's MST is limited. Moreover, despite the role of gender-based shame contributing to underutilization of treatment, and despite evidence that gender-tailored treatment approaches enhance engagement and outcomes, current MST best practices are not gender- tailored. Innovation: This study is the first known effort to refine and test an intervention tailored to men MST survivors, and to measure engagement and mental health recovery-oriented outcomes among this population. This study: 1) addresses a critical gap in research on men MST survivors, 2) focuses on improving treatment engagement through refining an innovative, recovery-oriented, and gender-tailored group therapy (the MMG) developed specifically for men MST survivors, and 3) assesses and enhances the intervention from a system- level perspective. Specific Aims: Building on promising pilot data, the Specific Aims are to: 1) revise the MMG treatment protocol with attention to factors that will support men's engagement and retention in mental health interventions; 2) test the intervention in a pilot randomized controlled trial and explore men's engagement as well as recovery-oriented and psychological outcomes; 3) understand process and treatment engagement factors and strategies for implementation, and prepare for a multisite trial. Merit proposals will be submitted in Year 3 to conduct a national mixed methods study of men's MST treatment approaches in VA, and in Year 5 to conduct a

multisite hybrid type 1 effectiveness-implementation trial of the MMG. Methodology: Aim 1: We will conduct two focus group interviews with previous MMG participants (n=8), semi-structured interviews with MST-focused clinicians and researchers (n=35) and convene an MST Expert Panel. Aim 2: We will randomize men MST survivors (n=32) to the MMG or Present-Centered Group Therapy at an outpatient mental health clinic. We will collect baseline, post-, and 6-month quantitative data assessing engagement as well as recovery measures (primary) and psychological symptoms (secondary). Aim 3: We will conduct qualitative interviews with participants and interventionists to assess process outcomes, return to the Aim 1 Expert Panel, and collaborate with operations partners to refine the protocol with system-level considerations. Aims 1 and 3 qualitative data will be analyzed using rapid qualitative analysis and thematic analysis. Aim 2 quantitative data will be analyzed using a linear mixed model. An integrated mixed methods analysis will be used to examine potential convergence of clinical and process outcomes. Next Steps/Implementation: This study was strategically designed to engage key stakeholders, particularly Veteran men, throughout, and results of this study will be shared with VA leaders in MST, mental health leaders, and Veterans. If outcomes are achieved, next steps will include testing the intervention in a multisite trial, with the long-term goal of system-wide implementation to expand appropriate gender-tailored health services for men with MST histories.

CRITIQUE 1

Overview of the Candidate's Training, Experience and Research Accomplishments:

- Jonathan Yahalom, PhD, completed his APA-accredited clinical psychology program at Duquesne University where he first worked in a Veterans' clinic and conducted ethnographic research on cultural factors in caregiving for dementia in Oaxaca, Mexico. He then completed a postdoctoral fellowship in integrative health and healing focused on mindfulness for Veterans and evidence-based trauma-focused therapies.
- Through his clinical work as a VA staff psychologist, he realized that military sexual trauma in men was under-reported, under-recognized and under-treated and developed a group therapy incorporating various evidence-based approaches. He also sought out expert mentors, such as Alison Hamilton, to better understand the factors contributing to the stigma and shame associated with MST in men.
- He has 12 peer-reviewed publications (11 first author) and has recently published four papers with his mentors.
- The research activities include the revision of an existing Men's MST Group (MMG) treatment, pilot testing the revised intervention compared to Patient-centered Group Therapy (placebo) and evaluating the perceptions of the therapy among patients and clinicians.
- This will be coupled with training in tailoring and adapting interventions, intersectionality, design and conduct of clinical trials, and integrated mixed methods.
- Dr. Yahalom will submit a Merit proposal to conduct a national survey and interview VHA providers (mixed methods study) about men's MST treatment approaches across the VHA and a second Merit proposal for a multisite RCT of the MMG intervention in Year 5.
- The proposed activities appear feasible in the requested five-year funding period, the mentors are experts and provide enthusiastic letters of support, and the environment and operations partnerships are strong.

Significance of Research Questions, Aims, or Hypotheses:

- Dr. Yahalom makes the case that MST in men Veterans is substantively different from the experience of women Veterans and that although MST disproportionately affects women, the absolute number of men suffering adverse consequences from MST is approximately the same (~80k).

- He highlights the importance of shame, stigma, and institutional betrayal and makes a theoretical link between these and certain established, evidence-based psychological treatment paradigms.
- He also convincingly argues that a gendered approach to treatment is missing from the current standard treatments available to men Veterans with MST and would likely be more effective. He makes the case that treatment is further complicated by overlapping identities (intersectionality).
- In this revision, he shifts the focus of the research activities slightly to examine more closely access to and engagement with treatment for MST among men Veterans.
- He also describes in more detail the process for refining the existing intervention and engaging Veterans in the process, increasing the likelihood of addressing the hypothesized issues of access and engagement.

Research Plan and Implementation Plan:

- Overall, the research activities appear feasible within the study time frame and appropriate to the overarching goal of the proposal. Several of the figures are too small to read. Many important details, e.g., domains of focus group and interview questions, heuristics of the adapted intervention, are only contained in appendices.
- The conceptual framework identified is intervention mapping, which seems appropriate for the narrow focus of revising, pilot testing, and evaluating the MMG intervention. It does not seem broad enough to address future directions of how the intervention fits into the context of the healthcare system or to inform the underlying issues affecting access to and engage with MST treatment by men. A more comprehensive and integrated conceptual framework could form the basis for the focus group and interview guides to ensure a systematic capture of issues.
- Aim 1a proposes focus groups with Veterans (2 groups x 4 Veterans) who previously completed the MMG. The lag between completion of the MMG and the focus groups is not clear but appears like it could be a long time (Dr. Yahalom states there are three cohorts/year). Also, four is a small 'group'- no mention is made of how no-shows would affect the focus group- but also a large proportion of patients (2/3 of each group). This raises concerns about feasibility of collecting these data.
- Aim 1b proposed provider interviews about treating men with MST.
- Aim 1c is a Delphi panel of experts identified from the providers interviewed in Aim 1b to 'advise on engagement strategies, MMG core components and outcome measures.
- Aim 2 is a randomized controlled trial, without explicit mention of blinding of the assessor, comparing the adapted MMG intervention to patient-centered group therapy (placebo). Two cohorts of 16 Veterans (8 in each group) will participate over 18 months. Dr. Yahalom will lead the MMG intervention for the first cohort and an unidentified provider will be trained to deliver the intervention for the second cohort. Training and assessment of treatment fidelity are addressed explicitly. The stated focus of the pilot is on feasibility and engagement, which are assessed in Aim 3; there are several measures of efficacy and its mediators assessed pre and post intervention and at 3 and 6 months after completion.
- Aim 3 focuses on eliciting participant and provider perspectives on the feasibility, acceptability, appropriateness and effectiveness through semi-structured interviews. These will be conducted immediately after completion or separation from the intervention. Potential participants who declined to participate will also be asked to complete a semi-structured interview.
- Dr. Yahalom proposes a national survey and interview mixed methods study of provider approaches to MST treatment for men Veterans as an IIR in year 3. This builds on experience and data from Aim 1, in particular.
- Dr. Yahalom proposes a multisite hybrid type 1 effectiveness study as an IIR in year 5. This builds on experience and data from Aims 2 and 3.

- Taken together, the aims will likely result in an improved MMG intervention but will still not produce conclusive evidence of efficacy of the adapted intervention. It is not clear that the next step after the CDA should be a hybrid type 1 effectiveness study in the absence of more compelling evidence of efficacy.

Innovation and Veteran Centricity:
- Adequate inclusion of Veterans and clinician stakeholders through focus groups, interviews, and inclusion in expert panels.
- The applicant makes a strong case that there is a gap in the treatment of men with MST and that his prototype treatment is an evidence-based approach that could fill that gap after the CDA.

Feasibility (including Sampling, Project Timeline and Staffing):
- The timing of recruitment of patient participants for Aim 1 is unclear. It appears that Dr. Yahalom is offering three courses of the MMG per year so recruiting patients from the most recent two cohorts could mean a period of 4-6 months since participation in the MMG. Also, with ~six patients per cohort, recruiting 8 participants from a total of 12 seems too optimistic. Finally, a focus group of four participants is at high risk of producing an actual group of two with just two no-shows. That would be an awkward group.
- The research activities, especially in year 5, seem slim for the 75% effort.
- The training program appears appropriate in scope and timing.

Career Plan:
- While the research activities are quite focused on the narrow topic of treatment for MST in men Veterans, Dr. Yahalom proposes to explore issues of intersectionality and identity, as well. Together, these may produce new avenues of exploration for Dr. Yahalom.
- His training foci are: (1) Tailoring and adapting interventions; (2) Intersectionality; (3) Design and conduct of clinical trials and health sciences research; (4) Integrated mixed methods.
- The training consists of an appropriate mix of formal didactics, experiential learning, and guided readings.
- The training plan should allow Dr. Yahalom to demonstrate mastery of mixed methods approaches, intervention development and testing. These skills will make Dr. Yahalom a capable independent investigator and an attractive collaborator.

Mentoring Plan:
- Dr. Yahalom has recruited a team of experienced and successful mentors led by Alison Hamilton.
- The mentors each contribute discrete expertise to the project and roles are clearly defined.
- There is a good plan for coordinating the mentorship.
- The proposed mentoring meetings and activities are appropriate.

Available Local Resources and Work Environment:
- The local environment at VA Greater Los Angeles Center for the Study of Healthcare Innovation, Implementation and Policy (CSHIIP) is blessed with an abundance of opportunities and support.
- The letters of support from the mentors, facility leaders, and director of the COIN are all very supportive.
- Dr. Yano, COIN director, indicates material support to the proposed work in years 4 and 5 and enthusiastic commitment throughout the duration of the study.

Adequacy of Response to Previous Feedback Regarding the Proposed Study:

- The response was complete to reviewer comments with noticeable enhancements and additions to the original proposal.
- The enhancements are generally effective, although the overall impression is that there was a slight shift in focus from a pilot clinical trial of efficacy to the same trial now decorated with attention to access and engagement. The conceptual framework was not expanded to incorporate these new foci. The shift highlights the lack of attention to the participant characteristics in the conceptual framework, as well. The heuristics buried in the appendix start to get at these concepts but they could be more thoroughly integrated into the main proposal.

Protection of Human Subjects from Research Risk:

- The human subjects section is very thorough and clear.
- Risks are explicitly described.
- Benefits are possible for those participants randomized to the MMG intervention.
- Risks are appropriately mitigated/managed.
- The knowledge that could be gained is important.

Inclusion of Women, Minorities and Children in Research:

- Women and men providers will be recruited and enrolled.
- Only men Veterans (not women Veterans) will participate in the project given the focus on men with MST.
- Under-represented minorities will be actively recruited and enrolled.

Budget & Period of Support (unscored):

- The budget appears adequate and appropriate.
- Resources from the CDA-2 mechanism are explicitly enhanced with contributions from the COIN.
- There is some question about the need for 75% protected time in year 5 given the limited CDA-2 research activities scheduled for that year.

Sharing Research Data (Data Management and Access Plan (DMAP)) (unscored):

- No concerns.

Overall Impression:

- Dr. Yahalom appears to be a committed VA clinician investigator with a strong interest and expertise in MST in men.
- HIs successful completion of the proposed work and training activities would likely allow him to transition to an independent investigator career with VA.

Key Strengths:

- Strong candidate with supportive and well-aligned mentoring team and local research and operations support.
- Interesting approach to MST in men, a high priority topic, with a track record of clinical and growing research experience in this area.
- Extensive qualitative exploration of clinical approach to treating MST in men including adaptation of an existing treatment developed by the PI.

Key Weaknesses:

- The conceptual framework does not appear to adequately encompass several key areas of relevance and focus of the proposal, especially patient and system characteristics that contribute to access and engagement in treatment for MST by men.
- The feasibility of recruiting patient participants for Aim 1 and collecting the focus group data as proposed is not convincing.
- The research activities in year 5 do not appear to justify continued 75% protected time.
- It is not clear that the data gathered in Aim 2 will be sufficient to move to a hybrid type 1 effectiveness study in year 5. The pilot is explicitly not powered to test efficacy of the intervention which would seem to be necessary to move on to study effectiveness.

CRITIQUE 2

Significance of Research Questions, Aims, or Hypotheses:
Dr. Yahalom's research plan and aims are compelling and address an unmet need within VA for a high priority group—men with MST. Specific aims of the research plan include: 1) revision of MMG protocol including attention to increased retention and engagement in mental health interventions; 2) conducting a pilot RCT comparing MMG to present centered group therapy; and 3) assessing pilot findings in preparation for a multi-site hybrid type 1 effectiveness implementation trial. These aims evolved from Dr. Yahalom's prior work in this area and are appropriate and sufficiently incremental.

Research Plan and Implementation Plan:
Dr. Yahalom's research plan is well written, logical, and convincing. As such some minor/moderate pertain to the qualitative components of study and timing of year 3 Merit:
Qualitative interviews seem more tailored to improve engagement for the MMG intervention but improvement in mental health engagement more broadly is noted as part of Aim 1. It seems like a missed opportunity to not ask about willingness to engage in CPT or PE or other MH services moving forward as part of Aim 1a and 3a. Otherwise, too, there is a mismatch between the research plan and aims.

What was the rationale for focus groups versus individual interviews in Aim 1a? Could a group format discourage some Veterans from speaking freely and would 30-45 minutes in a group setting allow sufficient time for everyone to weigh in?

Sample size for Aim 1b seems larger than necessary. Is significant variability within provider groups anticipated?

As part of Aim 3a, why will 32 completers of the intervention be interviewed? Why so large a sample? Why twice as many completers relative to those who drop out given the value of the latter group for informing engagement strategies down the road?

As part of Aims 1a and 3a, it would be helpful to ask Veterans how they perceive their functioning/well-being after completing the group relative to before---specifically overall functioning, perceived quality of life, as well as symptoms. These could be used in tandem with quantitative findings for a better understanding of the impact of the intervention on targeted outcomes.

An exclusionary criterion is completion of CPT, PE or the MMG group using chart review. How far back will the review go? Said differently, what is the farthest back time point for inclusion?

The timing of the Year 3 proposed Merit would potentially delay the planned Year 5 merit as the year 3 Merit is unlikely to have yielded sufficient data by that point in time.

Innovation and Veteran Centricity:
The proposed project is sufficiently innovative and Veteran centric given the target population and justification for importance of identity in intervention development for this group. Veteran feedback is also incorporated throughout the research plan.

Feasibility (including Sampling, Project Timeline and Staffing):
No concerns.

Career Plan:
Dr. Yahalom is a strong candidate for a CDA-2 given his training and recent endeavors. Specific training goals include: 1) tailoring and adapting interventions; 2) intersectionality; 3) design and conduct of clinical trials and health services research; and 4) integrated mixed methods. These are appropriate aims that align well with the research plan.

Mentoring Plan:
Dr. Hamilton will serve as primary mentor; Dr. Lang and Monteith will serve as co-mentos; and Drs. Der-Martirosian and Grant will serve as consultants. The team offers complementary but distinct areas of expertise and have a successful track record of health services research and mentorship.

Available Local Resources and Work Environment:
Excellent local work environment with sufficient resources.

Adequacy of Response to Previous Feedback Regarding the Proposed Study:
Dr. Yahalom was sufficiently responsive to prior feedback making changes to his application to better emphasize a health services approach in both the research plan and CDA portion of the application, inclusion of Veterans in the process of intervention refinement, and laying a compelling case for feasibility of recruitment. Some aspects of the qualitative component of the study remain unclear or insufficiently justified.

Protection of Human Subjects from Research Risk:
No concerns noted.

Inclusion of Women, Minorities and Children in Research:
No concerns. The target of the intervention is men.

Budget & Period of Support (unscored):
Appropriate.

Sharing Research Data (Data Management and Access Plan (DMAP)) (unscored):
No concerns noted.

Overall Impression:
Dr. Yahalom is a strong candidate for a CDA. The target of intervention is a high priority group for VA and one that would likely benefit from a gender tailored approach. The proposed intervention is promising with regard to preliminary data and potential for impact.

Key Strengths:

Intervention approach carefully developed from Dr. Yahalom's prior work and literature.
High priority group as target of intervention.
Strong candidate, mentorship team, and research environment.

Key Weaknesses:
Some details regarding the qualitative component of the study remain unclear or insufficiently detailed.
Concern about timing of the proposed Year 3 Merit—Dr. Yahalom will not have data from the Merit to adequately inform the proposed year 5 Merit.

CRITIQUE 3

Significance of Research Questions, Aims, or Hypotheses:
This well-written, well-argued application makes a strong case for the need for a gender-specific treatment for men with military sexual trauma (MST). The work strongly aligns with VA priorities and addresses an urgent, unmet need for Veterans. The current research plan incorporates a health system orientation that will enable Dr. Yahalom to emerge not only with a strong research base for improving care in the VA system but also with the training and skills to further these and future research endeavors.

Research Plan and Implementation Plan:
The research plan is well designed, well described, and comprehensive. It justifies its use of methods and addresses knowledge gaps in ways that will likely result in greater relevance and adoption of the MMG. The year 3 IIR is well explained and provides a strong next step that not only broadens understanding of the state of men's MST interventions in VA but also will help guide future work in this area. Throughout, Dr. Yahalom clarifies how the proposed research incorporates health services research methods. These methods support work that will potentially lead to changes in the health system at the local, regional, and national levels. My only comment about the research plan is that in Aim 1 it may be useful to think about what will be done if the recommendations from the round 2 focus groups and the Expert Panel do not agree.

Innovation and Veteran Centricity:
The study's focus on a neglected but high-need group, men who experienced MST, is highly innovative and directly aligned with Veteran need. The study now meaningfully involves Veterans in Aims 1 and 3 and now also leverages a strong health services research approach to ensure the intervention will be ready for greater system-wide implementation.

Feasibility (including Sampling, Project Timeline and Staffing):
No concerns. The study as described seems feasible. The timeline depicted is aggressive but, given the support of the mentoring team, would likely be achievable.

Career Plan:
The career plan is solid and focuses on building skills in support of a health services research career. The training activities align with the research plan and the year 3 IIR. The four training goals are mentioned in the research plan and are clearly relevant to the path Dr. Yahalom proposes pursuing.

Mentoring Plan:
The mentorship team is top tier. Dr. Yahalom has now authored five publications with his mentors. Given the excellent response to prior critiques, involvement of the mentorship team is evident. I have

no concerns about mentoring. Indeed, this team is poised to help Dr. Yahalom achieve the goals of this application.

Available Local Resources and Work Environment:
Strong local support. No concerns.

Adequacy of Response to Previous Feedback Regarding the Proposed Study:
The outstanding response to the previous reviews has greatly strengthened the proposal. Aims were revised to respond to issues raised, such as the incorporation of a health services research perspective and the inclusion of Veterans in the adaptation of the treatment. The inclusion of Vet Center mental health clinicians is a strength. Methods are now well described and justified. A conceptual framework guides the work. Concerns about mentor oversight are alleviated by the thorough response to prior critiques.

Protection of Human Subjects from Research Risk:
No concerns.

Inclusion of Women, Minorities and Children in Research:
This proposal rightly focuses on men. It makes an effort throughout to recruit underrepresented groups and specifically address issues of intersectionality.

Budget & Period of Support (unscored):
No concerns.

Sharing Research Data (Data Management and Access Plan (DMAP)) (unscored):
No concerns.

Overall Impression:
This proposal elegantly merges Dr. Yahalom’s clinical expertise with a larger career goal of engaging in meaningful health services research. Dr. Yahalom brings outstanding dedication and knowledge to the table about an area of critical importance. Men’s MST has been relatively neglected to date; this proposal aims to rectify this situation. The guidance and support of his mentorship team is evident throughout this revision and in the letters of support. Dr. Yahalom is an outstanding candidate for this award.

Key Strengths:

- Outstanding candidate who leverages his clinical background in the service of a proposed health services research career.
- Nationally renowned mentorship team whose involvement in the proposal is evident.
- Comprehensive response to prior critiques.

Key Weaknesses:
None worth noting.

MEETING ROSTER

Mentored Research
Health Services Research Parent IRG
Office of Research & Development
MRA0
08/22/2023 - 08/23/2023

CHAIRPERSON(S)

PUGH, MARY JO, PHD
RESEARCH HEALTH SCIENTIST
SALT LAKE CITY VAMC
PROFESSOR
DEPARTMENT OF GENERAL INTERNAL MEDICINE
UNIVERSITY OF UTAH
SALT LAKE CITY, UT 84132

RUDOLPH, JAMES L, MD
DIRECTOR
PROVIDENCE VAMC
CENTER OF INNOVATION IN GERIATRIC SERVICES
PROVIDENCE, RI 2908

MEMBERS

GRUBAUGH, ANOUK L, PHD
RESEARCH HEALTH SCIENTIST
CHARLESTON VAMC
ASSOCIATE PROFESSOR
MEDICAL UNIVERSITY OF SOUTH CAROLINA
CHARLESTON, SC 29403

HARTMANN, CHRISTINE W, PHD
RESEARCH HEALTH SCIENTIST
BEDFORD VAMC
RESEARCH PROFESSOR
UNIVERSITY OF MASSACHUSETTS LOWELL
BEDFORD, MA 01730

HELMER, DREW A, MD
DEPUTY DIRECTOR
HOUSTON VAMC
IQUEST
HOUSTON, TX 77030

HOGAN, TIMOTHY P, PHD
RESEARCH HEALTH SCIENTIST
BEDFORD VAMC
ASSOCIATE PROFESSOR
O'DONNELL SCHOOL OF PUBLIC HEALTH
UT SOUTHWESTERN MEDICAL CENTER
BEDFORD, MA 01730

HOU, JASON K, MD
CHIEF, GI & HEPATOLOGY
HOUSTON VAMC
ASSOCIATE PROFESSOR OF MEDICINE
BAYLOR COLLEGE OF MEDICINE
HOUSTON, TX 77030

LEE, SEI, MD
STAFF PHYSICIAN
SAN FRANCISCO VAMC
PROFESSOR OF MEDICINE
UNIVERSITY OF CALIFORNIA AT SAN FRANCISCO
SAN FRANCISCO, CA 94124

MATTOCKS, KRISTIN M, PHD
ACOS/RESEARCH
CENTRAL WESTERN MASSACHUSETTS VAMC
PROFESSOR
UNIVERSITY OF MASSACHUSETTS MEDICAL SCHOOL
LEEDS, MA 01053

MCANDREW, LISA M, PHD
HEALTH SCIENCE SPECIALIST
EAST ORANGE VAMC
WAR RELATED ILLNESS AND INJURY STUDY CENTER
EAST ORANGE, NJ 07018

MILLS, WHITNEY L, PHD
RESEARCH HEALTH SCIENCE SPECIALIST
PROVIDENCE VAMC
ASSOCIATE PROFESSOR
DEPARTMENT OF HEALTH SERVICES
BROWN UNIVERSITY
PROVIDENCE, RI 02908

ONG, MICHAEL K, MD, PHD
PHYSICIAN
GREATER LOS ANGELES VAMC
PROFESSOR IN RESIDENCE
UNIVERSITY OF CALIFORNIA LOS ANGELES
LOS ANGELES, CA 90073

ROUMIE, CHRISTIANNE L, MD
STAFF RESEARCH PHYSICIAN
NASHVILLE VAMC
PROFESSOR OF INTERNAL MEDICINE AND PEDIATRICS
VANDERBILT UNIVERSITY MEDICAL CENTER
NASHVILLE, TN 37212

SLATORE, CHRISTOPHER G, MD
PHYSICIAN-SCIENTIST
PORTLAND VAMC
PROFESSOR
OREGON HEALTH AND SCIENCE UNIVERSITY
PORTLAND, OR 97239

SOLIMEO, SAMANTHA, PHD
DIRECTOR OF OPERATIONS
IOWA CITY VAMC
ASSOCIATE PROFESSOR
DEPARTMENT OF GENERAL INTERNAL MEDICINE
UNIVERSITY OF IOWA
IOWA CITY, IA 52246

SOX-HARRIS, ALEX, PHD
RESEARCH CAREER SCIENTIST
PALO ALTO VAMC
PROFESSOR
DEPARTMENT OF SURGERY
STANFORD UNIVERSITY SCHOOL OF MEDICINE
PALO ALTO, CA 94304

STEPNOWSKY, CARL J, PHD
RESEARCH HEALTH SCIENTIST
SAN DIEGO VAMC
ADJUNCT PROFESSOR OF MEDICINE
UNIVERSITY OF CALIFORNIA, SAN DIEGO
SAN DIEGO, CA 92161

VOILS, CORRINE I, PHD
RESEARCH CAREER SCIENTIST
MADISON VAMC
PROFESSOR
SCHOOL OF MEDICINE & PUBLIC HEALTH
UNIVERSITY OF WISCONSIN
MADISON, WI 53705

SCIENTIFIC REVIEW OFFICER

SMALL, ROBERT
SCIENTIFIC REVIEW OFFICER
OFFICE OF RESEARCH & DEVELOPMENT
HEALTH SERVICES RESEARCH & DEVELOPMENT
VETERANS HEALTH ADMINISTRATION
DEPARTMENT OF VETERAN AFFAIRS
WASHINGTON, DC 20420

Consultants are required to absent themselves from the room during the review of any application if their presence would constitute or appear to constitute a conflict of interest.
